# Mid-term results of subcapital realignment of chronic slipped capital femoral epiphysis using surgical hip dislocation: a prospective case series

**DOI:** 10.1186/s10195-022-00676-1

**Published:** 2022-12-09

**Authors:** Ahmed Abdelazim Abosalem, Samy Abdel-Hady Sakr, Mohamed Kamal Mesregah, Ahmed Ibrahim Zayda

**Affiliations:** Department of Orthopaedic Surgery, Faculty of Medicine, Menoufia University, Shebin-El-Kom, Menoufia, Egypt

**Keywords:** Slipped capital femoral epiphysis, SCFE, Chronic slippage, Subcapital realignment, Surgical hip dislocation, Avascular necrosis

## Abstract

**Background:**

Slipped capital femoral epiphysis (SCFE) requires surgical treatment when diagnosed. The surgical management of moderate to severe SCFE remains an area of controversy among pediatric orthopedic surgeons. The severity of slippage, the viability of the femoral epiphysis, and the method of surgical management determine the long-term clinical and radiographical outcome. This study sought to evaluate the mid-term results of subcapital realignment of chronic stable slipped femoral epiphysis with open physis using surgical hip dislocation.

**Materials and methods:**

This study was a prospective case series of adolescents with moderate or severe degrees of chronic SCFE who had undergone subcapital osteotomy using the surgical hip dislocation technique. The Harris Hip Score (HHS) was used to assess functional outcomes at 6 years of follow-up. A HHS of ≥ 80 points was considered satisfactory. Postoperative radiological outcomes were evaluated using epiphyseal-shaft angles and alpha angles. Postoperative complications were observed.

**Results:**

This study included 40 patients, 32 (80%) males and 8 (20%) females, with a mean age of 14.1 ± 1.8 years. There was a statistically significant improvement in the mean HHS from 45 ± 12.3 preoperatively to 91.8 ± 11.6 points at 6 years of follow-up. The mean epiphyseal-shaft angle reduced from 60.5 ± 15.3° preoperatively to 10.3 ± 2.4° postoperatively, *P* < 0.001. The mean alpha angle reduced from 72.5 ± 10.1° preoperatively to 40.4 ± 6.4°, *P* < 0.001. Four (10%) patients showed femoral head avascular necrosis (AVN).

**Conclusions:**

Subcapital realignment of chronic SCFE can achieve satisfactory clinical and radiological outcomes, but femoral head AVN remains a risk.

***Level of evidence*** Level IV.

## Introduction

Slipped capital femoral epiphysis (SCFE) is a condition of displacement of the femoral head from the metaphysis through the physeal plate in adolescents [1, 2]. The incidence of SCFE around the world ranges from less than 1 to more than 7 per 100,000 children between 8 to 15 years old, depending upon gender and race [3].

The vast majority of SCFEs are idiopathic, and the slippage is usually attributed to mechanical factors such as thinning of the perichondrial ring complex with adolescent maturation and obesity [4–6]. Other causes include unidentified hormonal or biochemical factors that result in physis weakening, making it more susceptible to shearing forces, which in turn cause the actual slippage [5, 7]. SCFE can be classified using Southwick’s method to assess the degree of displacement of the epiphysis on the femoral neck by measuring the head-shaft angle on the slipped and the unaffected sides on anteroposterior and frog-leg lateral X-rays [8]. The degree of slippage is determined based on the difference in head-shaft angle between the slipped side and unaffected side into mild (< 30°), moderate (30–50°), and severe (> 50°) [1].

Depending on the duration of symptoms, SCFE can be acute (< 3 weeks) or chronic (> 3 weeks) [9].

The primary aim of treatment for SCFE is to stabilize the capital femoral epiphysis to the femoral neck to avoid further slippage. Other goals may include the closure of the capital femoral physis and the reduction of the epiphyseal displacement. Definitive treatment for SCFE includes in situ internal pinning and osteotomy through the apex or base of the femoral neck or intertrochanteric area, with fixation of the epiphysis to the femoral neck [10–12].

With in situ pinning of severe grades of SCFE, the hip remains deformed despite some remodeling, resulting in femoroacetabular impingement (FAI) and early development of osteoarthritis [13–15].

Realignment techniques for moderate or severe SCFE have been reported for subcapital, basicervical, and trochanteric levels [16–18].

If the proximal femoral physis is open, subcapital realignment can provide anatomical or near-anatomical correction of proximal femur deformities. This correction reduces the risk of early-onset osteoarthritis but increases the risk of osteonecrosis [19].

Resection of the metaphyseal callus reduces the tension on the retinacular vessels, reducing osteonecrosis risk [20–22].

The technique of safe hip surgical dislocation that was developed by Ganz et al. [23] encouraged Leunig et al. [22] to use this technique to do subcapital corrective osteotomy in SCFE, and was proposed as an option to restore the proximal femur anatomy.

The combined approach of subcapital realignment and surgical hip dislocation provides wide exposure of the femoral neck circumference, which facilitates the extension of the retinacular soft-tissue flap, femoral neck trimming, and safe reduction of the femoral epiphysis [22–24].

This study sought to evaluate the mid-term functional and radiological results of subcapital realignment of chronic SCFE combined with surgical hip dislocation.

## Materials and methods

### Patient selection

This study was a prospective case series of adolescents with moderate or severe chronic SCFE who had undergone subcapital realignment using the surgical hip dislocation approach from January 2014 to April 2015. All surgeries were performed by a single pediatric orthopedic surgeon. The Institutional Review Boards (IRB) and Ethics Committee of Menoufia University approved the study. Written consent for the procedure and possible complications was taken from the parents.

Inclusion criteria were patients between 8 and 17 years old who had moderate or severe degrees of chronic stable SCFE with open physis. A minimum of 6 years of follow-up were required for study inclusion.

Exclusion criteria were SCFE with other congenital or acquired hip deformities, SCFE in patients with known endocrinopathies, and a history of prior surgical interference. Those patients were excluded to avoid the effects of those variables on the results of the procedure.

### Preoperative evaluation

**Fig. 1:**
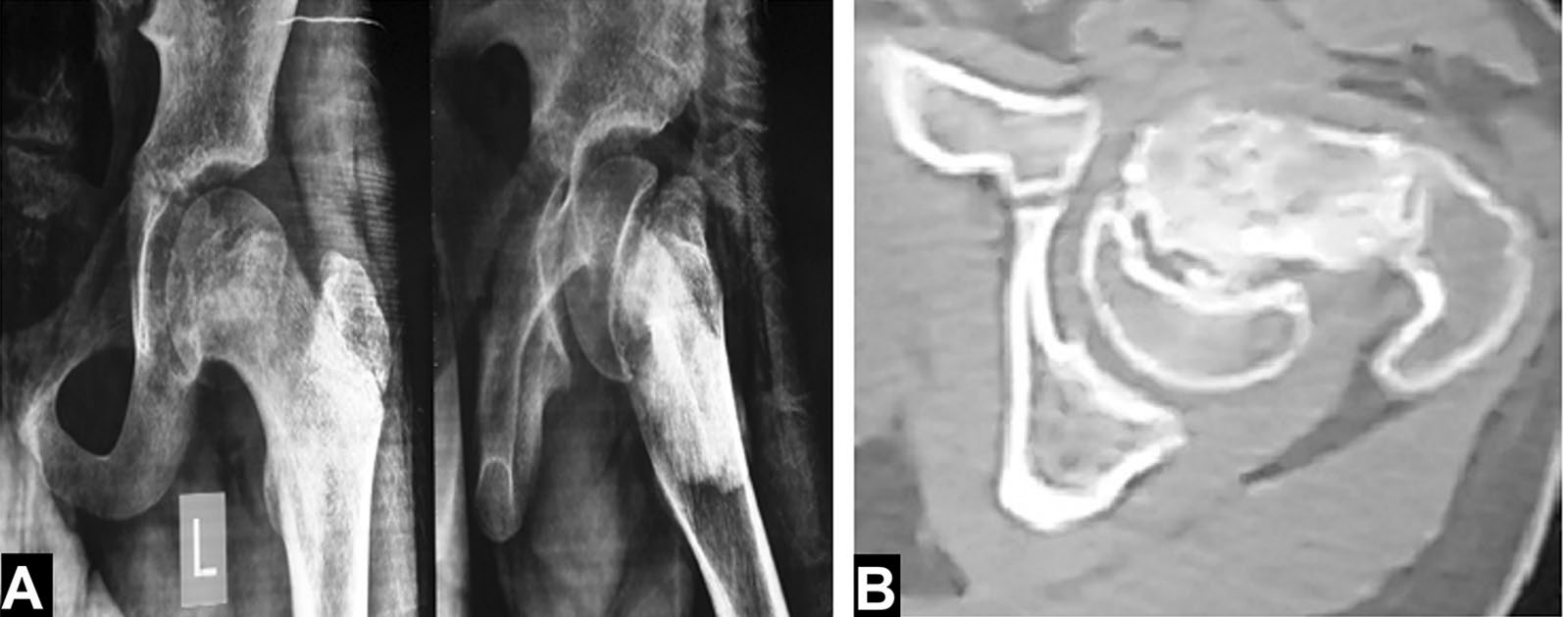
Preoperative images of a 13-year-old male patient with left chronic SCFE. **A** Plain X-rays (AP and lateral views). **B** CT axial cut

**Fig. 2:**
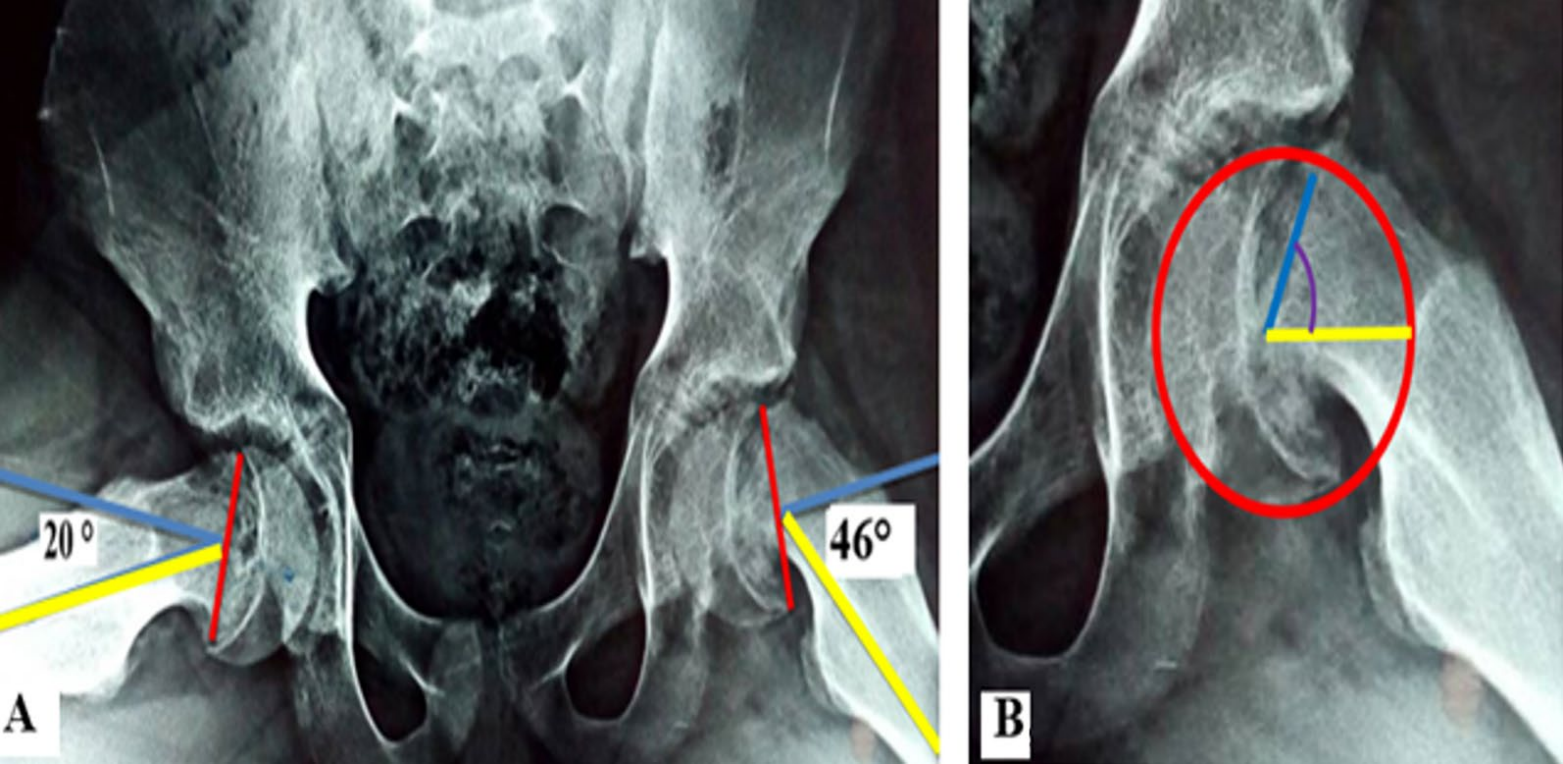
**A** Measurement of the epiphyseal-shaft angle bilaterally. **B** Measurement of the alpha angle

General and local examinations were performed to evaluate the body mass index (BMI), stability of slippage, hip joint range of motion, and preoperative limb length discrepancy (LLD). Plain X-rays of the slipped side were obtained. CT scan was used for the assessment of physeal closure and the attempted healing process in the proximal metaphysis that occurred with chronic displacement of the epiphysis; see Fig. 1. The epiphyseal-shaft and alpha angles were measured on the preoperative X-rays; see Fig. 2.

Preoperative laboratory investigations were done in the form of a complete blood count, a bleeding profile, liver and kidney function tests, and a hormonal assay in patients suspected of associated endocrinopathies (thyroid hormones, growth hormone, and testosterone levels).

### Surgical technique

Patients were given a third-generation cephalosporin injection 2 h before surgery, with the dose adjusted depending on the weight.

The procedure was done in the lateral position with the affected lower limb up. A straight skin incision was made over the greater trochanter and the fascia lata was incised.

**Fig. 3:**
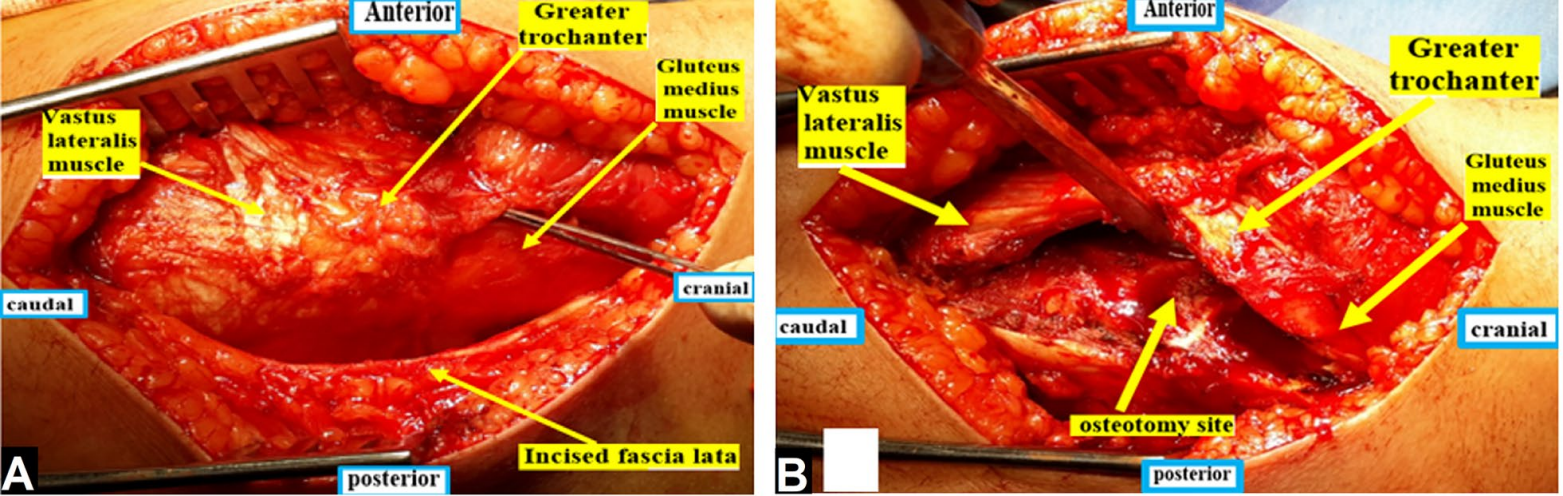
**A** Straight lateral skin incision and superficial dissection. **B** Greater trochanteric osteotomy

Greater trochanteric osteotomy with a maximum thickness of 1.5 cm was performed using an oscillating saw along a line extending between the posterosuperior edge of the greater trochanter and the posterior border of the vastus lateralis ridge. The trochanteric segment was mobilized anterosuperior by inserting a Hohmann elevator into the osteotomy gap after releasing the remaining tendinous parts of the gluteus medius from the stable trochanter and after subperiosteal dissection of the anterolateral part of the vastus lateralis from the femur; see Fig. 3.

Z-shaped capsulotomy was performed, where the vertical component started close to the anterosuperior corner of the stable trochanter and then directed proximally along the femoral neck axis. This was followed by an anterior capsular cut parallel to the intertrochanteric line and posterior capsulotomy along the acetabular rim.

The femoral head was temporarily stabilized by the insertion of two 2-mm Kirschner wires from the anterior aspect of the neck toward the center of the head to provide stability before dislocation.

**Fig. 4:**
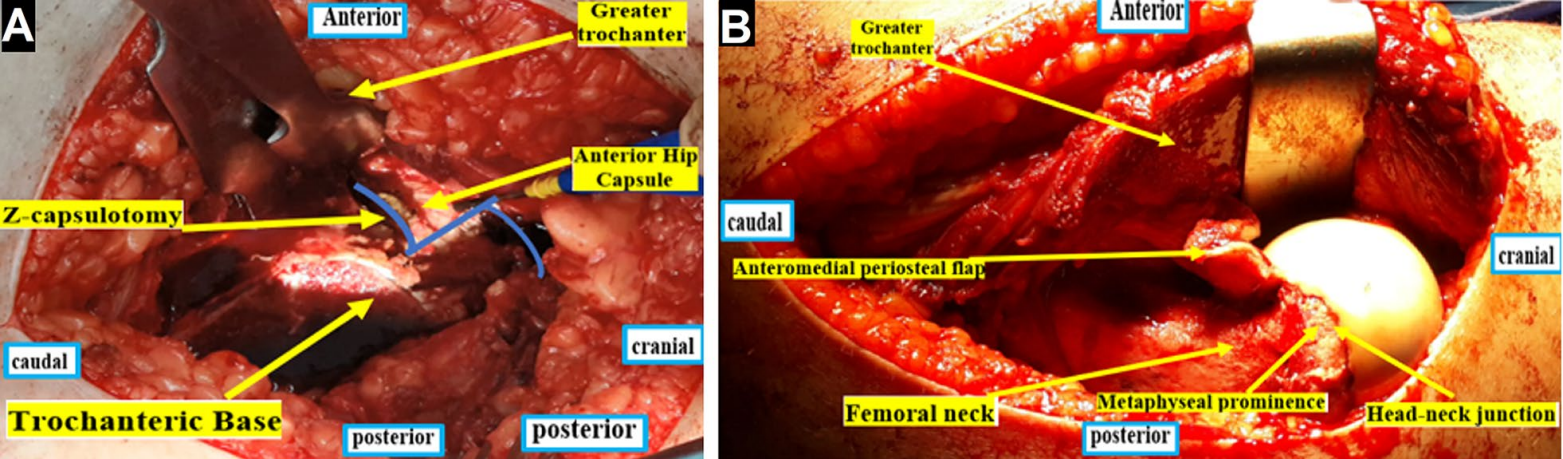
**A** Z-shaped capsulotomy. **B** Development of the periosteal-retinacular flap

Then the femoral head was dislocated by flexion, adduction, and external rotation of the hip after transection of the ligamentum teres using a scissor. After incising the periosteum along its axis, the retinacular flap was cautiously mobilized proximally with a periosteal elevator. The flap was extended distally to the lesser trochanter; see Fig. 4.

After removal of the transfixing K-wires, the femoral head was stepwise mobilized using an osteotome beginning from anterior. The femoral epiphysis with the attached periosteal-retinacular flap was gradually separated from the metaphysis, assisted by external rotation.

After complete separation of the epiphysis with its attached periosteal-retinacular flap, the metaphyseal callus was resected. A curette was used to clean the epiphysis from the growth plate tissue.

**Fig. 5:**
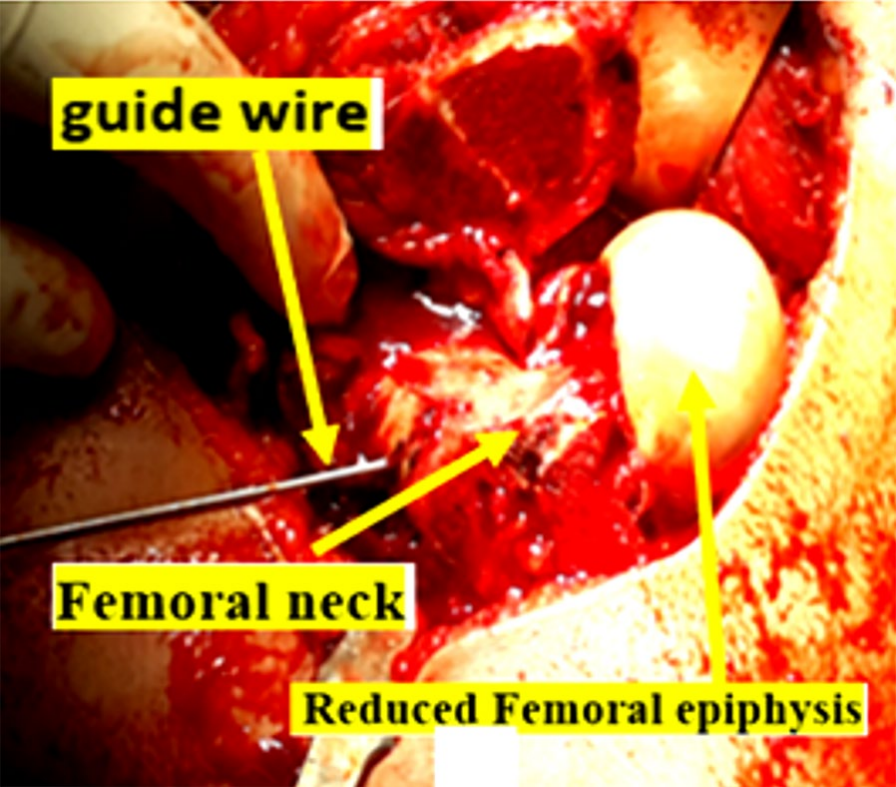
Reduction and provisional fixation of the head

Manual reduction of the epiphysis on the metaphyseal was made without any tension on the retinacular flap. In the case of a difficult reduction, further neck shortening was done to prevent tension on the retinacular vessels. The epiphysis was provisionally fixed by two or three threaded guide wires of the cannulated screws; see Fig. 5. The threaded guide wires were advanced till they were flush with the articular surface and were checked by fluoroscopy.

Definitive fixation of the epiphysis was done using two 6.5-mm partially threaded cannulated screws. The length of the screws was 5 mm shorter than the actual measure of the threaded guide wires. The correct alignment of the epiphysis was checked by fluoroscopy by restoring the epiphyseal-metaphyseal anterior offset and correcting the epiphyseal-shaft angle after epiphyseal reduction to about 145° on the AP view and 10° on the frog-leg lateral view [8, 25].

Perfusion of the epiphysis was monitored periodically by making a 2-mm drill hole in the epiphysis and observing the resulting bleeding. The anterior periosteum and the posterior retinacular flap were reattached using tension-free sutures, and the capsule was closed without tension. Fixation of the osteotomized greater trochanter with two 4.5-mm cortical fully threaded screws was done in a more distal position (0.5–1 cm) to place the abductors under proper tension. A suction drain was inserted, followed by closure of the fascia lata, subcutaneous tissue, and the skin.

### Postoperative care and functional assessment

**Fig. 6:**
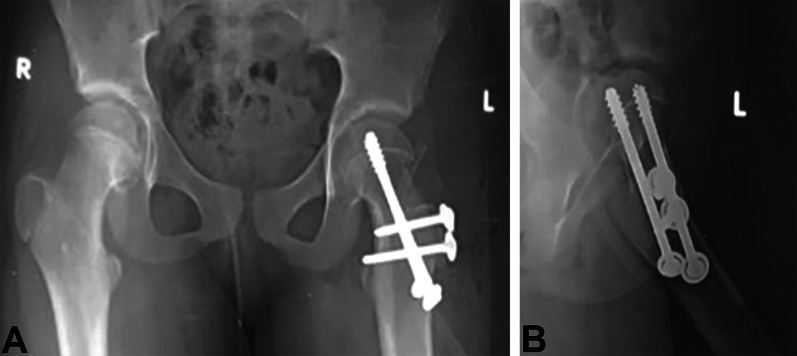
Immediately postoperative X-rays. **A** AP view. **B** Lateral view

Postoperative plain X-rays were done; see Fig. 6. Passive hip range-of-motion exercises were commenced during the hospital stay. The suction drain was removed after 24 h.

**Fig. 7:**
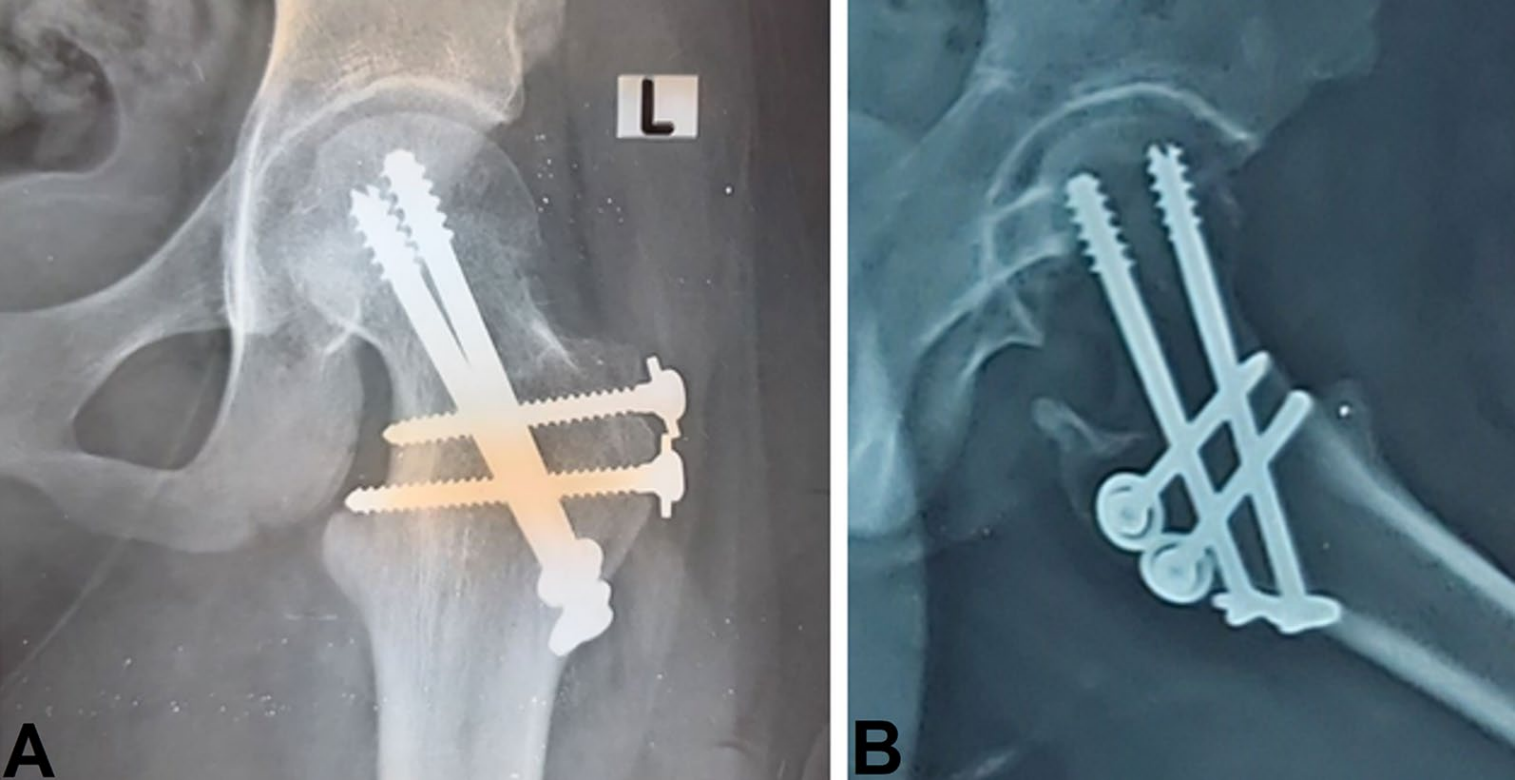
Six-year follow-up X-rays. **A** AP view. **B** Lateral view

Follow-up in the outpatient clinic was done with an assessment of wound status and suture removal after 2 weeks. Moreover, evaluation of the radiological union was performed to start weight bearing and physiotherapy after 3 months. Another evaluation was performed after 1 year for clinical and radiological assessment and for the detection of complications. The final clinical and radiological evaluation was done after 6 years of follow-up; see Fig. 7.

Clinical outcomes were assessed with the Harris Hip Score (HHS) [26]. A satisfactory outcome was considered to be a HHS of ≥ 80 points. Measurement of the epiphyseal-shaft angle and alpha angle was done postoperatively.

### Statistical analysis

At the end of the study, the data were collected, tabulated, and statistically analyzed by IBM SPSS (Statistical Package for the Social Sciences) version 23. The chi-square test and Wilcoxon test were utilized for comparative statistical analysis when appropriate. The significance level was set at* P* values of less than 0.05.

## Results

### Demographics and baseline characteristics

A total of 40 patients were included, 32 (80%) males and 8 (20%) females, with an average age of 14.1 ± 1.8 (range, 11–17) years. Thirteen (32.5%) patients had a moderate degree of slippage, with a mean slip angle of 40.1 ± 6.3° (range, 32–50°), and 27 (67.5%) patients had a severe degree, with a mean slip angle of 61.4 ± 13.9° (range, 52–88°). The left side was affected in 29 (72.5%) patients, and the right side was affected in 11 (27.5%). Eight (20%) patients had contralateral SCFE of a mild degree that was managed by pinning in situ. The mean duration of symptoms was 5.5 ± 1.6 (range, 2.5–9) months. The mean BMI was 25.7 ± 4.2 (range, 21–34) kg/m^2^. The mean preoperative LLD was 1.7 ± 0.7 (range, 1–3) cm. The mean preoperative HHS was 45 ± 12.3 (range, 23–56) points. The mean preoperative epiphyseal-shaft angle was 60.5 ± 15.3° (range, 42–98°), and the mean preoperative alpha angle was 72.5 ± 10.1° (range, 57–98°); see Table 1.

**Table 1:** Demographics and baseline characteristics of the included patients

Characteristic	Value (*n *= 40)
Age, years (mean ± SD)	14.1 ± 1.8
Gender (n, %)	
Males	32 (80%)
Females	8 (20%)
Degree of slippage (*n*, %)	
Moderate	13 (32.5%)
Severe	27 (67.5%)
Side (*n*, %)	
Left	29 (72.5%)
Right	11 (27.5%)
Duration of symptoms, months (mean ± SD)	5.5 ± 1.6
Preoperative body mass index (BMI) (mean ± SD)	25.7 ± 4.2
Preoperative Harris Hip Score (HHS), points (mean ± SD)	45 ± 12.4
Preoperative limb length discrepancy (LLD), cm (mean ± SD)	1.7 ± 0.7
Preoperative epiphyseal-shaft angle, degrees (mean ± SD)	60.5 ± 15.3
Preoperative alpha angle, degrees (mean ± SD)	72.5 ± 10.1

### Functional and radiological outcomes

The mean length of hospital stay was 5 (range 3–7) days. The mean follow-up period was 6.6 ± 0.5 (range, 6–7.3) years. The mean LLD was reduced postoperatively to 0.3 ± 0.5 (range, 0–1.5) cm,* P* < 0.001. The mean follow-up HHS showed a statistically significant improvement to 91.8 ± 11.6 (range, 64–100) points, *P* = 0.0014. Excellent results were achieved in 34 (85%) patients, fair results in 2 (5%) patients, and poor results in 4 (10%) patients; see Table 2. Follow-up HHS showed a significant negative correlation with the BMI (*r*_s_ = − 0.729 with *P* = 0.0011). Follow-up HHS showed no other significant correlations with other preoperative risk factors (age, preoperative slip angle, and preoperative alpha angle); see Table 3.

**Table 2:** Grading of functional results based on the follow-up Harris Hip Score

Result	Score	*n *= 40	Percentage (%)
Excellent	90–100	34	85
Good	80–89	0	0
Fair	70–79	2	5
Poor	< 70	4	10

**Table 3:** Correlation between follow-up HHS and preoperative risk factors

Variable	Follow-up HHS	
	*r*_s_ (correlation coefficient)	*P* value
Age	− 0.359	0.121
BMI	− 0.729	0.0011^*^
Preoperative slip angle	− 0.217	0.358
Preoperative alpha angle	− 0.124	0.604

^*^P < 0.05 is statistically significant

The mean postoperative epiphyseal-shaft angle was 10.3 ± 2.4° (range, 6–16°), *P* < 0.001. The mean postoperative alpha angle was 40.4 ± 6.4° (33–49°), *P* < 0.001.

### Complications

**Fig. 8:**
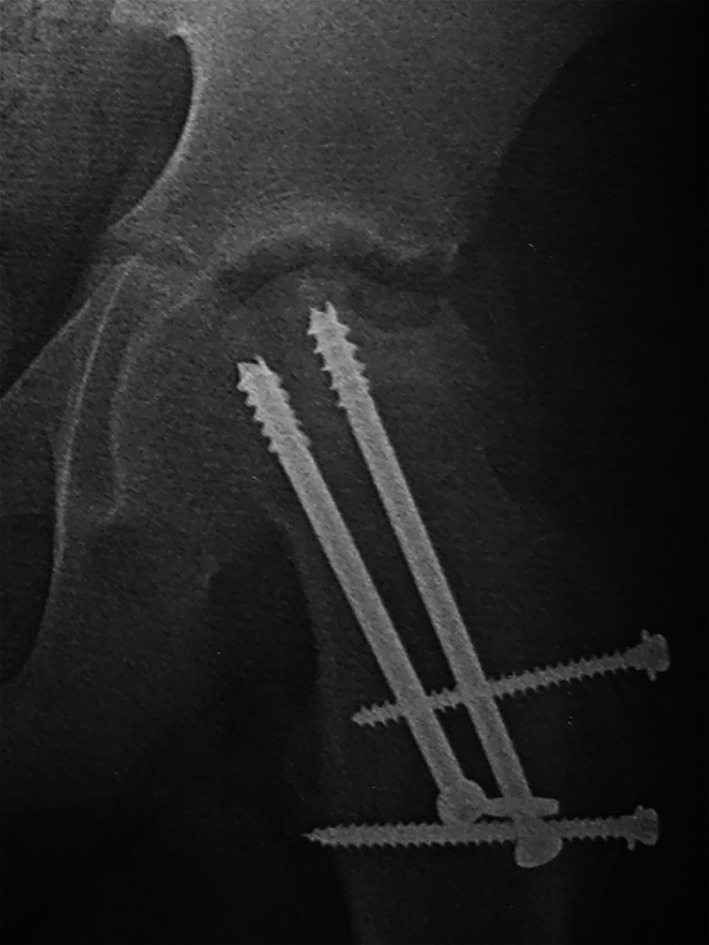
Plain X-ray (AP view) showing segmental avascular necrosis of the femoral head

Complications were reported in 6 (15%) patients. Four (10%) patients showed AVN of the femoral head that was diagnosed in the follow-up X-rays by the presence of subchondral radiolucency with the crescent sign that indicates subchondral collapse. Two of them developed segmental AVN 9 months after surgery; see Fig. 8. After 6 years of follow-up, the 2 patients had mild pain that was controllable by analgesics. The other 2 patients developed total AVN 6 months after the surgery. Clinically, the patients had moderate to severe pain with limitations of ordinary activities. Those were managed by total hip replacement. Two (5%) patients developed postoperative hip dislocation 1 week after surgery. This was attributed to excess shortening of the femoral neck to decrease the tension on the retinacular vessels. They were managed by closed reduction with an anti-rotational cast and hip abduction brace for 4 weeks to limit hip joint motion.

## Discussion

Once diagnosed, SCFE is usually treated surgically to prevent further progression of the slippage, correct deformity, and avoid complications such as chondrolysis and AVN. There is a significant debate about the best method of treating SCFE, especially for moderate and severe degrees [1, 4]. Many years ago, in situ fixation was considered the gold standard in the treatment of SCFE, with a relatively low rate of complications [27, 28].

In our study, patients with chronic moderate or severe degrees of slippage were treated by subcapital realignment using the surgical hip dislocation approach. The mean HHS improved significantly from 45 preoperatively to 91.8 at the latest follow-up. There was a significant improvement in the epiphyseal-shaft angle, alpha angle, and LLD. The functional results were excellent in 85% of patients, fair in 5% of patients, and poor in 10% of patients.

Ziebarth et al. [19] reported excellent results according to the HHS, with a mean score of 99.6 points. Slongo et al. [29] reported that the mean HHS was 99 points, and 87% of the patients had satisfactory postoperative outcomes. Othman et al. [30] reported that the mean final follow-up HHS was 86.2 points, and the functional outcome was excellent and good in 75% of the patients, fair in 10%, and poor in 15%. Ebert et al. [31] reported good results in 73.33% of the patients (HHS > 80 points). In a study by Masquijo et al. [32], the mean HHS was 76.3 points. Excellent results were achieved in 35% of the patients, good in 30%, fair in 10%, and poor in 25% [32].

In this study, the incidence of AVN was 10%. The incidence of AVN varied in previous studies. Ziebarth et al. [19] reported that none of the patients developed osteonecrosis. The incidence of AVN was 2% in a study by Tannast et al. [25], 5% in a study by Slongo et al. [29], 5% in a study by Othman et al. [30], 6% in a study by Khalil et al. [33], and 26% in a study by Ebert et al. [31]. Masquijo et al. [32] reported that the overall incidence of AVN was 47.6% and that a higher incidence of AVN developed in unstable slips (53%) than in stable ones (33%).

Cosma et al. [34] used two 6.5-mm cannulated screws for fixation of the epiphysis and two 3.5-mm screws for fixation of the osteotomized trochanteric segment, with no incidence of implant failure. Tannast et al. [25] reported improved stability after using 4.5-mm screws instead of pins in the epiphyseal and trochanter fixation used in their initial patients. Ebert et al. [31] reported no implant failure after fixing the epiphysis with two 6.5-mm cannulated screws and fixing the greater trochanter with two 4.5- or 6.5-mm screws. In our study, definitive fixation of the epiphysis was done by two 6.5-mm cannulated, partially threaded cancellous screws, with no implant failure or re-slippage. Fixation of the greater trochanter was done by two 4.5-mm fully threaded cancellous screws. One patient showed a partial loss of reduction of the greater trochanter with slight proximal migration. We think that the 4.5-mm fully threaded cancellous screws used in the fixation of the trochanteric fragment should engage two cortices to provide adequate stability.

Accadbled et al. [35] concluded that a 35° slip angle is the threshold level beyond which FAI is more frequent, because remodeling at the head-neck junction is not to be expected after that degree of slippage. Therefore, with mild degrees of slippage, in situ fixation would be an appropriate method of treatment, as the remodeling potential is high. In contrast, higher degrees of slippage are best managed with capital realignment using surgical hip dislocation [19, 21]. Some authors prefer initial in situ fixation and secondary osteochondroplasty or subtrochanteric osteotomy in cases with moderate degrees of slippage to avoid capital realignment complications, especially avascular necrosis [35].

Subcapital realignment using surgical hip dislocation corrects the SCFE at the deformity level, restores the normal head and neck offset, and eliminates the FAI and secondary chondrolabral injury caused by the metaphyseal prominence [21, 36]. Yet, this procedure is more invasive and technically demanding than in situ pinning, with a higher incidence of AVN [37].

Limitations of this study include the relatively small number of patients and the non-comparative nature of the study, with the absence of a control group.

## Conclusion

Subcapital realignment of chronic moderate or severe degrees of SCFE using surgical hip dislocation provides highly significant clinical and radiological improvements in the HHS, epiphyseal-shaft angle, and alpha angle. However, it carries a risk that AVN of the femoral epiphysis will develop.

## Data Availability

The dataset analyzed in this study is available from the corresponding author on request.
